# Consistent unmet needs and fragmented care in Rett syndrome: insights from a multi-country European caregiver study

**DOI:** 10.1186/s13023-026-04651-5

**Published:** 2026-10-17

**Authors:** Tom Pulles, Becky Jenner, Mariëlle van den Berg, Pedro Rocha, Angela De Martini, Kees Chamberlain, Harish Grandjean

**Affiliations:** 1 Acadia Pharmaceuticals GmbH, Zug, Switzerland; 2 Rett Syndrome Europe, Luxembourg City, Luxemburg; 3 Rett UK, Luton, United Kingdom; 4 Nederlandse Rett Syndroom Vereniging, Utrecht, Netherlands; 5 Asociacion Española de Síndrome de Rett, Valencia, Spain; 6 Charles Rivers Associates, Zurich, Switzerland

**Keywords:** Rett syndrome, Caregiver burden, Unmet needs, Rare disease management, Multidisciplinary care

## Abstract

**Background:**

Rett syndrome is a rare neurodevelopmental disorder associated with functional impairments that emerge in early childhood and persist throughout life. Despite improved understanding of its genetic basis and natural history, therapeutic options remain limited, and care frameworks across Europe are highly variable. This study aimed to characterize the lived experiences, unmet needs, and evolving challenges faced by individuals with Rett syndrome and their caregivers across multiple European countries.

**Results:**

Sixty caregivers across eight countries completed five-day diaries, and thirty participated in in-depth interviews. The sample represented children (36%), adolescents (42%), and adults (22%) with Rett syndrome; 97% were female. Difficulty using hands (reported by 88% of caregivers) and difficulty communicating (78%) were the most frequently observed symptoms. Daily activities were heavily affected, with symptoms impacting 59% of activities for children, 61% for adolescents, and 38% for adults. Some comorbidities increased with age, including scoliosis (45% in children vs. 92% in adults) and pneumonia (54% in adults). Care demands were substantial, with caregivers of adults spending an average of 41 hours per week providing emotional support alone, along with high time requirements for feeding (20 hours), washing and bathing (8 hours), and administering medication (5 hours).

**Conclusions:**

Caregiver experiences across Europe reveal a consistent pattern of unmet needs, high symptom burden, and fragmented, poorly coordinated care for individuals with Rett syndrome. The changing needs across the lifespan underscore the importance of anticipatory, multidisciplinary care models and improved transition pathways into adulthood. As participants were recruited through patient advocacy groups, the study population may represent a comparatively informed and healthcare-engaged subgroup of caregivers, highlighting that unmet needs and care challenges across the broader RTT caregiver community may be even greater among families with less access to advocacy networks and specialized support resources. These findings highlight the need for system-level changes and the development of comprehensive, standardized care frameworks for Rett syndrome across Europe.

## Background

Rett Syndrome (RTT) is a rare neurodevelopmental disorder characterized by neurological impairment that begins in infancy and continues into adulthood. Development in classical RTT individuals is apparently normal until symptoms appear at 6–18 months, typically including deceleration of head growth, loss of fine motor movements and expressive language skills, and the development of distinctive repetitive hand movements such as wringing, washing, or clapping [1]. The clinical phenotype of RTT is broad, and clinical diagnosis is based on these typical symptoms alongside supportive criteria, including breathing difficulties, impaired sleep, intense eye communication, and musculoskeletal abnormalities like scoliosis [1]. The severity of symptoms is also variable, and a more severe clinical status is associated with worse physical quality of life (QOL) [2].

RTT is associated with pathogenic variants of *MECP2*, whose product, MeCP2, is highly expressed in the brain and required for the development and function of the nervous system [3, 4]. *MECP2* is located on the X chromosome; therefore, RTT affects females almost exclusively. Mutations in males can result in a wide range of phenotypes and may often cause embryonic lethality [5]. There is a wide range of *MECP2* mutations that can drive the development of RTT, while atypical RTT can also be caused by pathogenic variants in CDKL5 or FOXG1 [6, 7]. As such, genetic-based diagnosis remains challenging, and clinical diagnosis is typically through presentation of symptoms [1]. While the genetic and neurobiological background of RTT is now well understood, there is currently no approved therapeutic treatment for RTT in Europe. However, a new treatment based on a derivative of Insulin-like Growth Factor 1 (IGF-1) was approved by the FDA in 2023 for the treatment of RTT [8].

Outcomes for individuals with RTT have improved through advances in diagnosis and increased recognition of factors affecting mortality. A 2014 study found the likelihood of survival at 37 years was 59.8%, while a publication the following year found that survival at 45 years was greater than 70% [9, 10]. As such, healthcare professionals and caregivers must navigate an evolving landscape of needs throughout a person’s lifetime, from childhood to adulthood. Disease management focuses on minimizing the impact of symptoms and comorbidities, including seizures and mobility issues [11, 12]

Individuals with RTT are typically dependent on caregivers for all activities throughout their lives. This places a significant emotional, physical, and financial burden on parents, families, and other caregivers [13–16]. The impact of having a child with a disability on the mental health of caregivers is widely appreciated [17–20], and studies have suggested an increased risk of anxiety and depression in the mothers of RTT individuals [13, 15]. Typically, caregivers of RTT individuals have to make significant alterations to all aspects of their lives, and interviews with caregivers have highlighted how financial challenges related to full-time care can exacerbate health-related anxieties [21].

Understanding factors that influence burden of illness (BOI) is essential for effective disease management. Work that seeks to reduce BOI must focus on the impact on individuals with RTT and their caregivers throughout the RTT journey. Recent work has highlighted core clinical features of RTT, as well as organizational and environmental factors as important drivers of overall BOI [22, 23]. Caregivers of children with rare diseases have highlighted how a lack of awareness among healthcare professionals (HCPs) can act as a barrier to access to care, and how fragmentation of care increases the burden placed on them to navigate multiple care roles [24].

The impact of symptoms and management approaches on BOI and QOL for individuals with RTT and their caregivers throughout diagnosis, disease management, and the transition into adulthood remains poorly understood. To develop an understanding of the factors impacting patient experience, we gathered survey information from caregivers to assess the impact of symptoms on a pan-European sample of RTT individuals. We also conducted in-depth interviews with RTT caregivers to understand their experiences of the RTT journey, including diagnosis and ongoing care, as well as the impact on their physical and emotional well-being.

## Methods

### Caregiver diaries

Caregivers for an individual with Rett syndrome (*N* = 60) were recruited from Germany (*N* = 8), Italy (*N* = 8), UK (*N* = 8), France (*N* = 6), Denmark (*N* = 8), Poland (*N* = 8), Spain (*N* = 7), and the Netherlands (*N* = 7), with the support of patient advocacy groups. Inclusion criteria were adult caregiver with at least 1 year of experience as primary caregiver for an individual with Rett syndrome, including accompanying them to medical appointments, discussing management options with healthcare providers, and helping manage needs related to daily living. RTT individuals were grouped by age: child (2–11), adolescent (12–17), adult (18+).

Diary questionnaires were developed based on key issues raised by caregivers and existing literature, as well as any gaps identified therein. The diary was reviewed by selected individuals representing Rett patient associations from each country included in the research to ensure its appropriateness and relevance. Rather than adopting existing validated instruments, the diary and interview tools were designed to address the specific priority questions of this research. While established measures of caregiver burden and symptom severity exist in the broader rare disease literature, their application would have constrained the scope of inquiry and precluded exploration of aspects of the RTT experience that remain poorly characterized. The questionnaire was developed in coordination with patient advocacy groups, and was informed by existing instruments, drawing on their frameworks while adapting content to reflect the specificity of this research on RTT.

The survey protocol was reviewed and validated by Advarra’s Institutional Review Board (IRB) using the Department of Health and Human Services regulations found at 45 CFR 46.104(d)(2) and was determined to be exempt from IRB oversight. All methods were performed in accordance with the WMA Declaration of Helsinki. All participants consented to the collection and use of their personal information, acknowledged their understanding of their right to privacy, and consented to participate in the research.

Caregiver diaries were completed on five days over the course of a week. On each day, caregivers answered questions regarding symptoms experienced by the RTT individual, as well as their own emotions during this time. Caregivers also answered a different set of questions regarding aspects of the RTT experiences on each of the five days. These aspects were diagnosis and key milestones, communication, physical mobility, food and diet, and access to care.

For questions in multiple-choice format, the frequency of each answer was calculated as a percentage of the number of participants in each group. For questions related to symptom experience that were assessed daily over five days, experiencing a symptom on any day was coded as ‘present’ for analysis. For scales of severity, the mean severity was calculated for each group. Between-group analyses were performed based on the country and age of the RTT individual. Between-group differences in categorical variables were assessed using contingency table analyses: age group comparisons were analyzed using chi-square tests of independence; country comparisons employed Fisher’s exact tests due to small expected cell frequencies. Between-group differences in ranked variables were assessed using Kruskal-Wallis tests. Multiple testing correction using Benjamini-Hochberg was conducted within distinct question families: each individual day of questions and the set of daily questions were treated as separate testing families. All statistical analysis was completed in RStudio (version 2024.12.1 + 563).

### Caregiver interviews

Caregiver interviews (*N* = 30) were also undertaken with participants from Germany (*N* = 4), Italy (*N* = 4), UK (*N* = 4), France (*N* = 4), Denmark (*N* = 3), Poland (*N* = 4), Spain (*N* = 4), and the Netherlands (*N* = 3). Interviews consisted of 60-minute, one-to-one video interviews with specific on-screen stimuli to help guide discussion. Topics covered were “Life before diagnosis”, “Living with Rett syndrome”, “Patient community”, and “Expectations from new treatments”. Guide for interviewers and on-screen stimuli are provided in supplementary materials.

## Results

### Participant demographics

The study included a sample of RTT individuals, including children (36%), adolescents (42%), and adults (22%) (Table 1). Highlighting the X-linked nature of RTT-driving mutations, 97% of RTT individuals in the study were female. The majority of caregivers included in the study were parents (96%), most commonly between 36 and 55 years of age (75%) (Table 2).

**Table 1:** RTT patient demographics. Adol = adolescent

	By age group			By country								Total
	Child	Adol	Adult	UK	FR	DE	IT	ES	DK	NL	PO	
**n**	22	25	13	8	6	8	8	7	8	7	8	60
**Sex**												
Female	22	24	12	7	6	8	8	7	7	7	8	58
Male	0	1	1	1	0	0	0	0	1	0	0	2
**Time since RTT diagnosis**												
Less than 1 year ago	1	0	0	0	0	0	1	0	0	0	0	1
1–2 years ago	6	0	0	1	1	0	0	0	0	3	1	6
3–5 years ago	5	0	0	1	0	0	1	0	2	0	1	5
6–10 years	10	9	0	2	0	4	2	4	2	1	4	19
More than 10 years ago	0	16	13	4	5	4	4	3	4	3	2	29
**Ability to communicate**												
Significant difficulty	16	15	5	4	6	5	0	4	8	4	5	36
Reasonable ability	6	10	8	4	0	3	8	3	0	3	3	24

**Table 2:** Caregiver demographics. Adol = adolescent

	By age group			By country								Total
	Child	Adol	Adult	UK	FR	DE	IT	ES	DK	NL	PO	
**n**	22	25	13	8	6	8	8	7	8	7	8	60
**Age**												
26–35 years old	6	0	0	1	0	0	0	0	1	1	3	6
36–45 years old	10	13	1	2	3	1	2	4	5	3	4	24
46–55 years old	6	11	4	3	2	5	4	3	2	1	1	21
56–65 years old	0	1	5	1	1	1	1	0	0	2	0	6
Over 65 years old	0	0	3	1	0	1	1	0	0	0	0	3
**Care Status**												
Care for their child with RTT	22	25	11	7	6	8	8	7	8	6	8	58
Care for someone with RTT at a residential facility	0	0	2	1	0	0	0	0	1	1	0	2
**Time as caregiver**												
1–2 years	4	0	0	1	0	0	1	0	0	2	0	4
2–3 years	1	0	0	0	0	0	0	0	0	1	0	1
4–5 years	3	0	0	0	1	0	0	0	0	0	2	3
6–10 years	11	1	0	2	0	3	2	0	4	0	1	12
More than 10 years	3	24	13	5	5	5	5	7	4	4	5	40

Adol = adolescent

### RTT symptoms and impact on quality of life

To understand the impact of RTT symptoms on everyday activities and quality of life, caregivers reported the frequency and severity of symptoms each day throughout the study (Figs. 1a and 1b). Difficulty using hands (88%) and difficulty communicating (78%) were the most frequently reported symptoms across all age groups and countries, with no statistically significant differences observed between age groups or countries. However, some trends in the frequency of symptoms were observed across age groups. Anxiety, difficulty with mobility, difficulty swallowing, mood swings, difficulty communicating, and repetitive facial expressions were all most commonly reported in children and least commonly reported in adults (Fig. 1a). Conversely, active seizures were rarely reported during the 5 diary days in children (9%) and experienced more frequently among adults (26%). The occurrence of comorbidities related to RTT was also assessed (Fig. 1c). Scoliosis was highly prevalent among adults (92%) and less frequently noted in children (45%). Similarly, pneumonia was mostly commonly reported as affecting adults (54%).

**Fig. 1:**
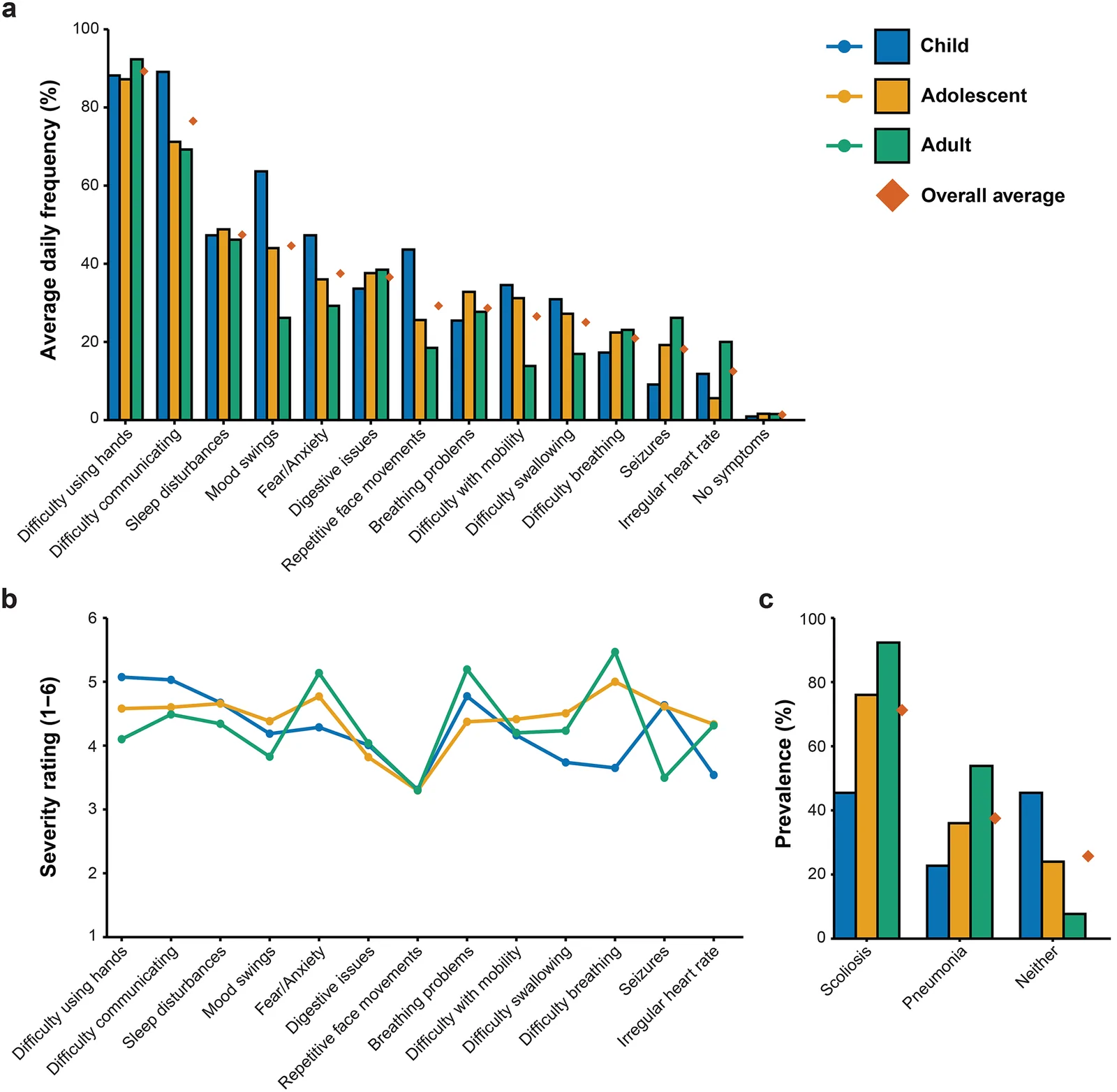
Symptoms and comorbidities experienced by the patients. **a**) average frequency with which a range of RTT-related symptoms were reported across the 5  days. **b**) average severity of the symptoms that were reported across the 5 days. Answers were provided on a 1–6 scale, with 1 representing ‘not bothersome at all’ and 6 representing ‘extremely bothersome. **c**) average prevalence of known RTT comorbidities (scoliosis and pneumonia) in the patients

Symptoms were frequently reported to affect many aspects of daily activity, particularly those related to mobility, including using hands, walking/running, and bathing/showering (Fig. 2a). On average, the frequency with which activities assessed were impacted was 59% and 61% for children and adolescents, respectively, and only 37.6% for adults. However, it is possible that these data reflect lower participation in some activities in adults overall.

**Fig. 2:**
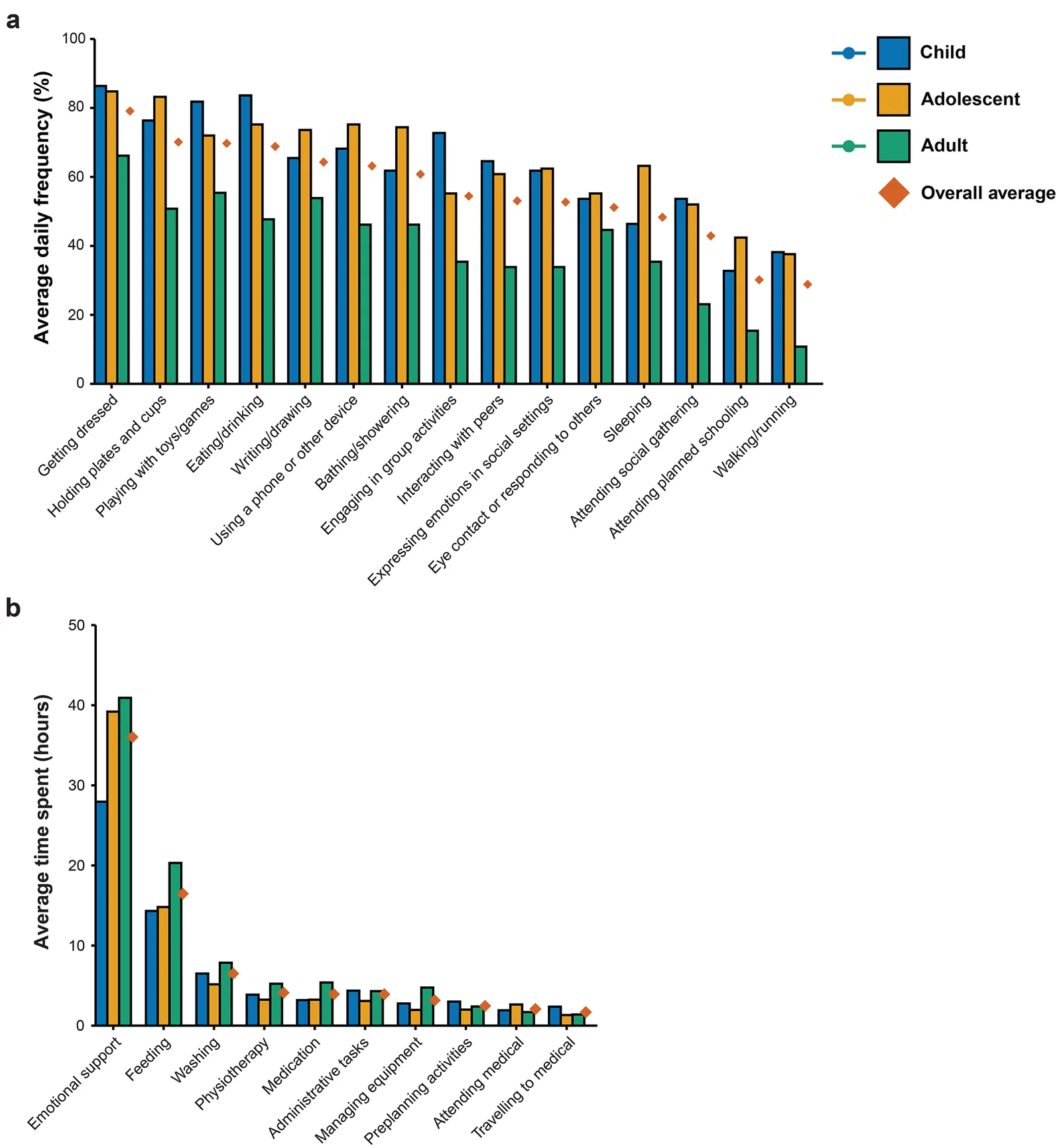
Impact of RTT on daily activities and time spent on caregiving activities. **a**) average frequency with which a range of everyday activities were impacted by RTT-related symptoms across the 5 days. **b**) average number of hours that the caregivers spend on different activities per week

Caregiver interviews also highlighted the severe impact that RTT symptoms can have on the physical health of caregivers, with one participant noting, “*Imagine moving a person with scoliosis who weighs nearly as much as yourself, and who is nearly as tall as you.*” While the physical impacts on caregivers highlight the level of assistance RTT individuals require for mobility, caregivers reported spending the most hours of care in “providing emotional support”. Time spent on many caregiving activities was highest in adults, including washing and bathing (7.8 hours per week), feeding (20.3 hours per week), administering medication (5.4 hours per week), helping with physio exercises (5.2 hours per week), and managing the equipment of the patient (4.8 hours per week), as well as providing emotional support and care (40.9 hours per week) (Fig. 2b). Time spent on activities was estimated by participants, and there is likely to be overlap among caregiving activities. Caregiver interviews consistently pointed to the burden of caregiving and the impact it has on their daily activity. One caregiver noted *“Free times can be counted perhaps on the fingers of one hand, even half a hand. You stop being husband and wife, you become 24 hours mother and father.”*

### Changing needs of RTT individuals

Trends in the frequency of symptoms and complications experienced at different age groups highlight the changing needs of RTT individuals. Trends observed in participant reporting of the use of aids for mobility and communication further highlighted potential differences. Wheelchair use was highest among adults (77%) and lowest among children (45%) (Fig. 3a), as was the use of feeding tubes (38% in adults and 18% in children) (Fig. 3b).

**Fig. 3:**
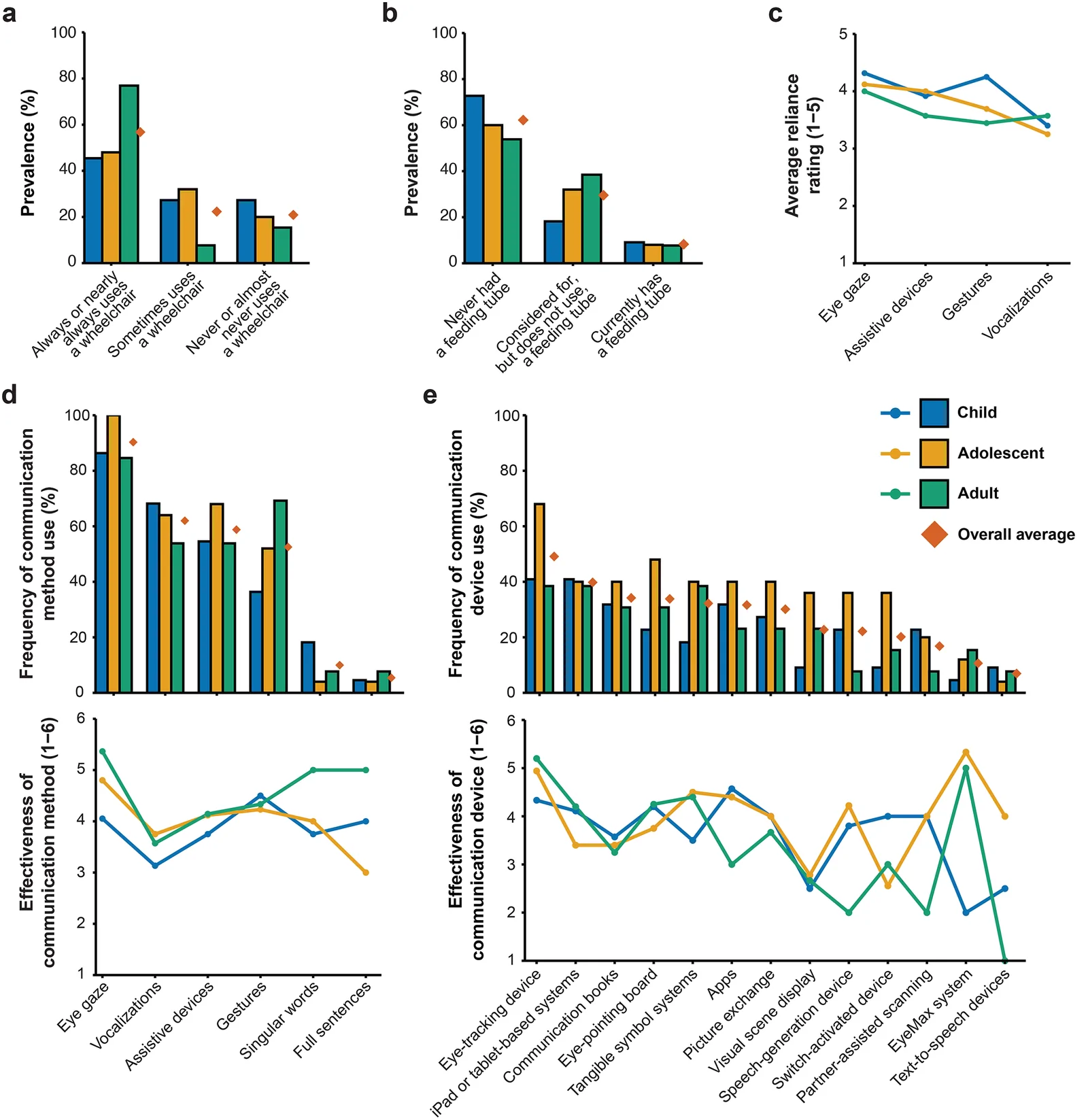
Abilities and needs of the RTT patients. **a**) average wheelchair usage by the patients. **b**) average feeding tube usage by the patients. ‘Considered for a feeding tube’ includes RTT patients that have discussed the use of a feeding tube with their healthcare advisors, but are not currently using one. **c**) change in dependency upon communication techniques by the patients over time. Answers were scored from 1–5, where 1 represented ‘has become less used,’ 3 represented ‘no change,’ and 5 represented ‘has become more used.’ higher scores indicate greater reliance on the associated technique in order to communicate. **d**) average frequency with which the patients use different communication techniques and a line plot (bottom) showing the average effectiveness of each technique. Effectiveness was scored on a 1–6 scale, where 1 represented ‘not effective at all’ and 6 represented ‘extremely effective.’ **e**) average frequency with which the patients use different devices to assist their communication (top) and average effectiveness of each device (bottom). Effectiveness was scored on a 1–6 scale, where 1 represented ‘not effective at all’ and 6 represented ‘extremely effective’

Difficulty communicating was frequently reported as an issue impacting QOL for RTT individuals and their caregivers, and aids are commonly used to assist in communication. Eye gaze was the form of communication with the highest rating of reliance (Fig. 3c). Vocalization (not distinguishable words) was the most common form of communication among children (68%), and adolescents (64%), while gestures were the most common form of communication among adults (69%) (Fig. 3d). Speech was less frequently reported as a primary form of communication, reflecting the severity of communication issues faced by RTT individuals. Augmented and Alternative Communication (AAC) systems, which help individuals express themselves using symbols, text, and text-to-speech, were the most commonly used assistive tools for communication. The use of assistive devices tended to be more frequent in children and adolescents than in adults (Fig. 3e). This is supported by trends in reported efficacy of such devices, which were rated 4.6 and 4.4 in children and adolescents, respectively, and 3.0 in adults. Eye tracking systems were rated as highly effective by caregivers of adults with RTT. Caregiver interviews also highlighted issues related to communication, with one participant reporting: “*I believe that* [communication] *in my opinion is the largest problem she has, she can’t express herself. She can’t respond appropriately to the situation, in the right time, so that we can understand what she want*s.”

The capacity of RTT individuals to eat and drink can have a significant impact on their well-being and QOL. Participants consistently reported difficulties with eating, with only 54% of caregivers reporting that the individual is always able to swallow (Fig. 4a). The inability to drink independently was highest in adults (50%), while still prominent in children (44% can sometimes or rarely/never drink independently) (Fig. 4b). Difficulty eating is accompanied by a range of gastrointestinal issues, of which constipation was reported most frequently (Fig. 4c).

**Fig. 4:**
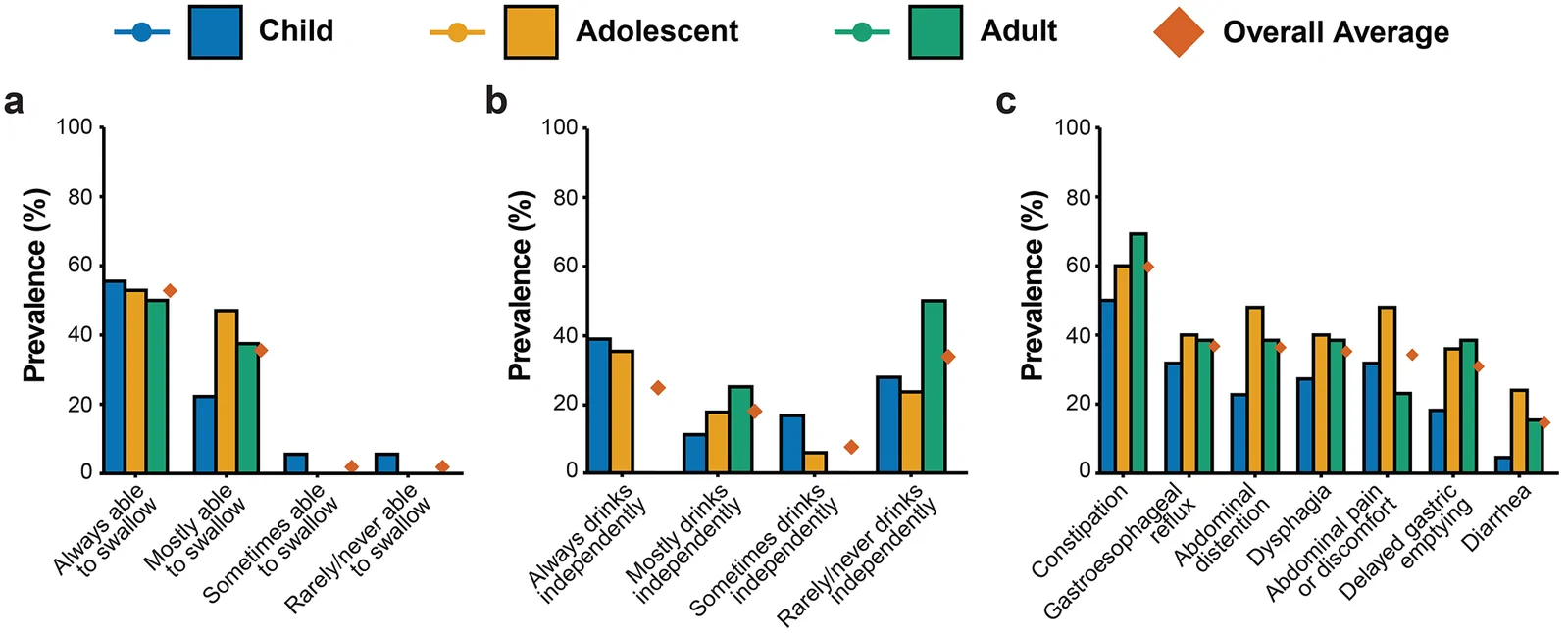
RTT patients’ ability to swallow and the prevalence of gastrointestinal symptoms. **a**) average ability of the patients to swallow food. **b**) average ability of the patients to consume drinks. **c**) average prevalence of different gastrointestinal symptoms in the patients

### Access to care

Trends in age-specific differences in symptoms, challenges, and the use of aids, highlight the changing needs for RTT individuals through aging and major milestones. One caregiver interviewee noted *“Moving from a children’s hospital to an adult facility is quite jarring. Finding a suitable hospital took a long time. We also had to transition from children’s day-care to semi-adult day-care, which took about two years. Medically, things became quite challenging, and we’ve faced many difficulties, including with paperwork and insurance.”* This also highlights the importance of access to appropriate specialized care. Caregivers’ experiences of access to care were also assessed through diary questionnaires, which revealed a wide variety in the type and specialty of healthcare professionals (HCPs) seen regularly (Fig. 5a), with the average number of specialists seen being 9. The majority of RTT individuals had seen a dietitian or nutritionist, highlighting the impact of nutrition and gastrointestinal issues (Fig. 5b). The majority had a physician as their primary point of contact (Fig. 5c), while most commonly they were only seen once a year (Fig. 5d). The most common specialties for primary physicians were pediatric neurologists for children (39%) and general practitioners for adults (25%).

**Fig. 5:**
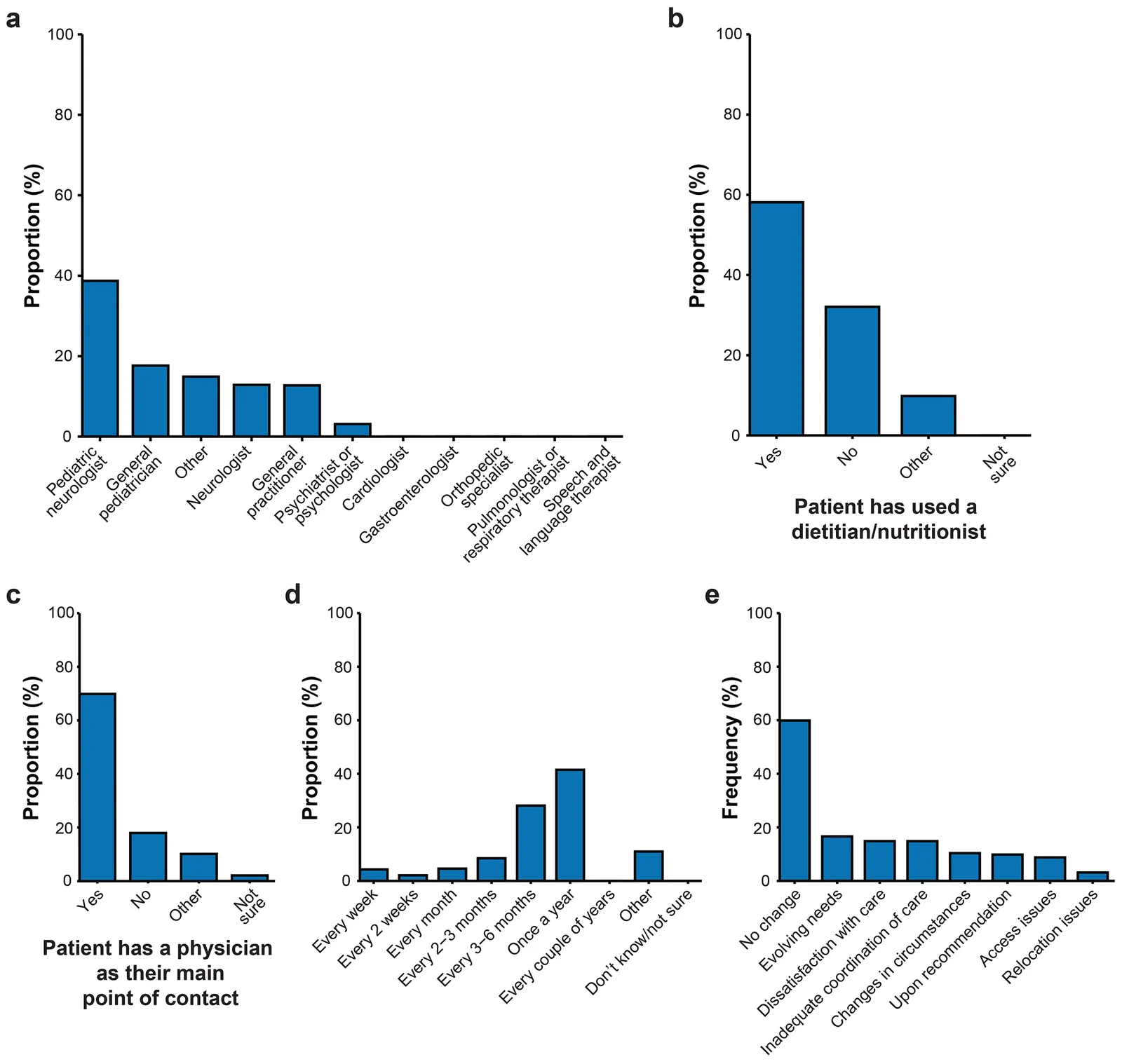
RTT patients’ access to care and the relationship with physicians. **a**) specialties of the main physicians that care for the patients. **b**) overall proportion of patients who have worked with a dietitian or nutritionist. **c**) overall proportion of patients that have a physician as their main point of contact. **d**) frequency with which the patients see their main physician. **e**) proportion of patients that have not changed their main physician (‘no change’) as well as the reasons for which other patients have changed their main physician

Caregivers emphasized the difficulties they have faced in accessing the proper care. 87% of participants reported having to change their primary physician, with the most common reported reasons including dissatisfaction with care (15%), inadequate follow-up or coordination of care (15%), and evolving needs (17%) (Fig. 5e). Only 3% of RTT patients access a sleep specialist, despite sleep being a highly disruptive issue. The most commonly seen healthcare practitioners were physical therapists, highlighting consistent issues with symptoms related to mobility (Fig. 6).

**Fig. 6:**
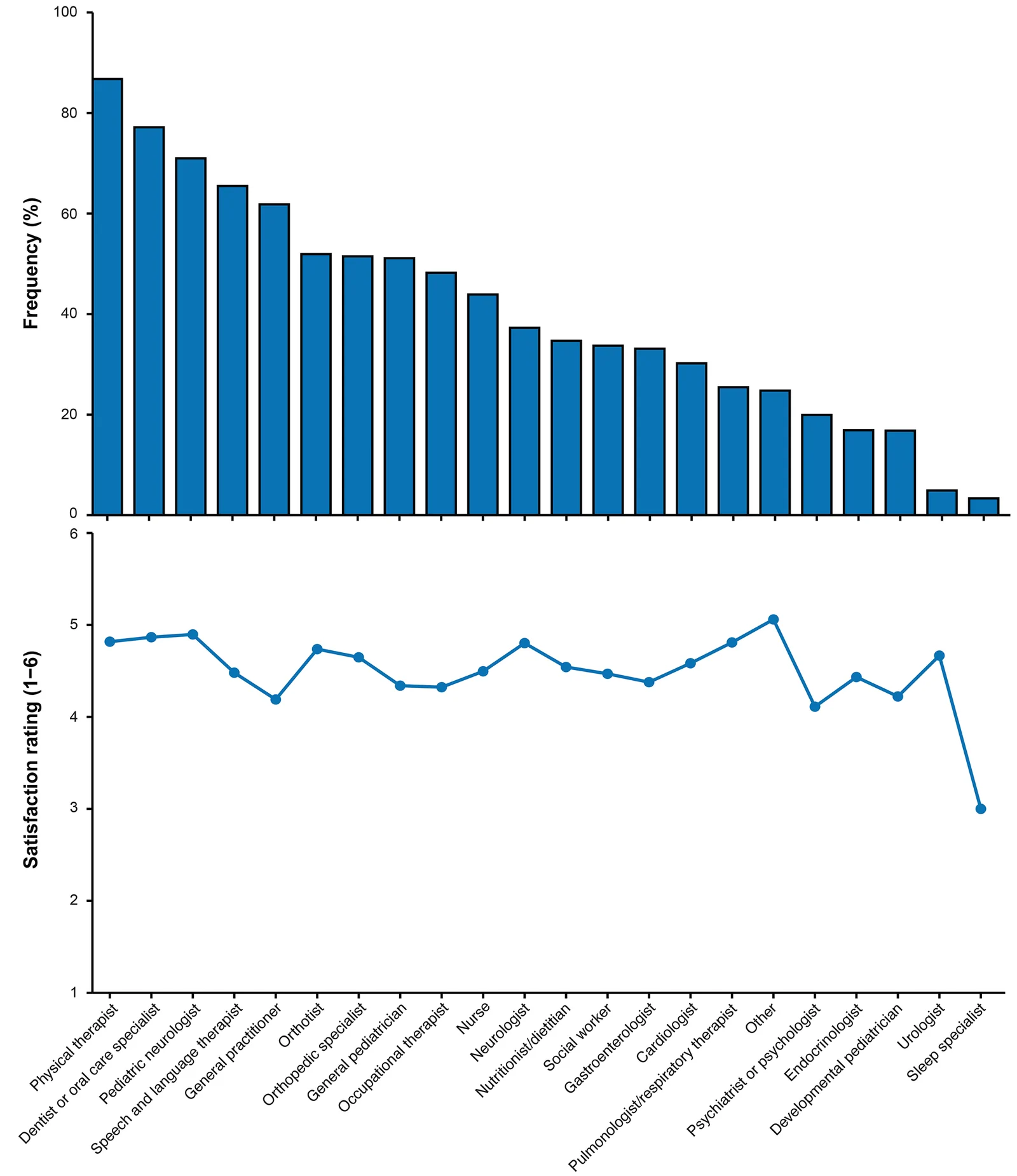
Healthcare professionals involved in the care process. Average frequency with which different healthcare professionals (HCPs) are regularly seen by the patients (top) and average level of satisfaction that the patients have for each type of HCP (bottom). Satisfaction was scored on a 1–6 scale, with 1 representing ‘not satisfied at all’ and 6 representing ‘extremely satisfied’

Caregivers consistently highlighted fragmentation of care more broadly as a major hurdle to accessing care: “*Unless you go in a specialist center for Rett, although even there it’s hard to achieve synergy, in my opinion. It needs teamwork, something like a control booth to pull the strings of all the actors revolving around the syndrome”*. This is exacerbated in the transition from childhood to adulthood: “*Moving from a children’s hospital to an adult facility is quite jarring. Finding a suitable hospital took a long time. We also had to transition from children’s day-care to semi-adult day-care, which took about two years. Medically, things became quite challenging, and we’ve faced many difficulties, including with paperwork and insurance.”*

Care was also reported to be geographically fragmented with only 3% of participants reporting that they received all their care at the same location, and the most common answer regarding locations of care was “very few in one location” (30%) (Fig. 7a). Obtaining accurate information following diagnosis was also emphasized as a barrier to providing effective care. 68% of participants reported sometimes receiving conflicting information from HCPs (Fig. 7b), with the most common source of conflicting information being the doctor’s office (59%) (Fig. 7c). Instead, caregivers highlighted the importance of Rett-specific support groups as a source of information: “*[The Rett association] were the ones who help us understand the disease through various workshops about genetics, about the different techniques for taking care of the child. We had training sessions on genetics and mutations, explaining to us, what is a gene? Why are there genetic problems?”.*

**Fig. 7:**
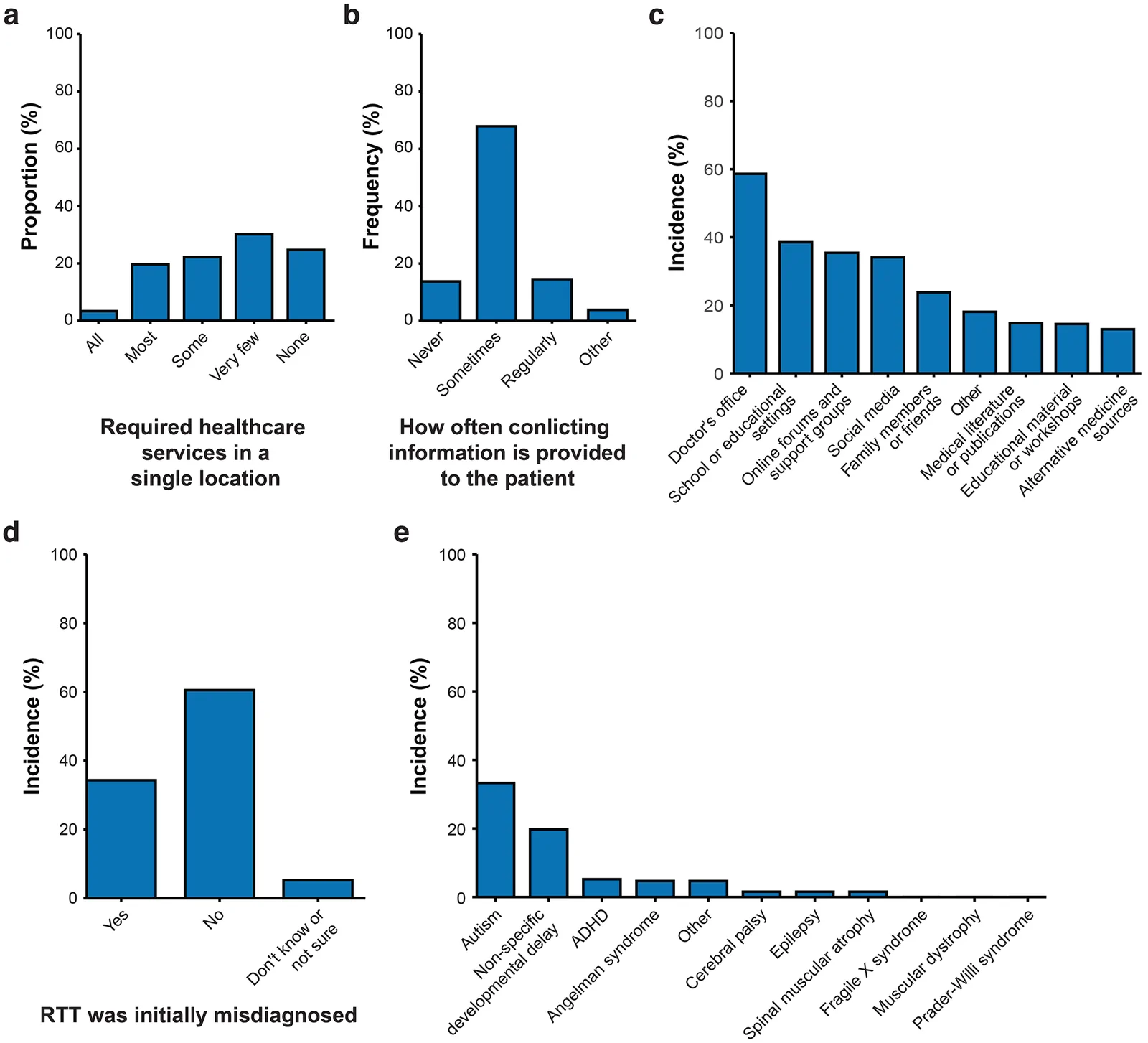
Access to healthcare services and the distribution of conflicting information. **a**) a bar chart showing the location of healthcare services that are necessary for the patient, indicating the proportion of services that are located in the same place. **b**) a bar chart showing the frequency with which patients/carers have received conflicting information from two different healthcare professionals. **c**) a bar chart showing the different sources of conflicting information and the proportion of patients that have been affected. **d**) a bar chart showing the proportion of RTT patients who were initially misdiagnosed with another condition. **e**) a bar chart showing the different conditions that the RTT patients were initially misdiagnosed with

Caregivers also highlighted difficulties in obtaining an accurate diagnosis following initial symptoms as a major hurdle. 34% of participants reported that the RTT individual for whom they care had been misdiagnosed following the appearance of symptoms (Fig. 7d), most commonly with autism (33%) (Fig. 7e). This was reported in cases where participants reported clear symptoms (“*I didn’t understand how it could take so long to diagnose my daughter, when you see that my daughter’s Rett syndrome is textbook”*) and where symptoms were less clear (“*the doctors kept referring us further simply because apart from delayed psychomotor development, nothing else was visible. So we just kept bouncing from doctor to doctor, from hospital ward to hospital ward”*).

## Discussion

Improving the QOL and reducing the BOI for RTT requires a comprehensive understanding of the factors that most significantly impact RTT individuals and their caregivers. RTT presents early in life, and as such, understanding the experiences of RTT individuals and their caregivers at different ages can reveal the changing nature of these factors to help deliver the most appropriate care at the right time. Furthermore, RTT is a rare condition with poorly defined frameworks for care at different milestones of life and in the transitions between them. As such, this study assessed the experiences of caregivers from across Europe to identify consistent issues in care. The data presented here contribute to a limited amount of currently available literature regarding the management of RTT. One recent study investigated the RTT burden of illness across several countries (USA, United Kingdom, Italy, Germany, and Australia) and found no significant differences between age and country [22].

Here, we focused on the impact of symptoms on individuals with RTT as experienced and reported by parents/caregivers, as well as the burden these symptoms place on caregivers. We developed bespoke diary and interview tools to capture RTT-specific findings. While we acknowledge that this approach limits direct comparison with existing BOI literature, the use of study-specific instruments rather than any standardised scales enables discovery-oriented flexibility rather than quantitative comparability. In line with previous research [23, 24], caregivers consistently reported that symptom burden has a major impact on daily quality of life. We found that difficulty using hands and difficulty communicating were the most frequently reported symptoms, with issues related to mobility consistently impacting RTT individuals. We also found no significant differences between countries. This may suggest that the experiences and needs of RTT individuals and their caregivers are consistent across a diverse range of healthcare systems and settings.

Several methodological limitations of this study were considered when interpreting these findings. RTT is a rare condition, with a prevalence estimated at approximately 1 in 10,000 female births, and this inherently constrains the sample sizes achievable in any real-world study of this population [25]. As such, the absence of statistically significant differences between countries may reflect true homogeneity in caregiver experiences across European healthcare systems, but may also result from insufficient statistical power to detect meaningful differences where they exist. Recruitment through individual country patient advocacy groups is an effective way to connect with rare disease patients and their caregivers, and ensure they are represented fairly. However, this can also introduce bias; families connected to advocacy networks are more likely to be informed about available resources and more engaged with the care system than those without such connections. As such, the experiences reported here may reflect a comparatively well-supported and informed subgroup of caregivers, while unmet needs and care challenges across the broader RTT community may be even greater among families with less access to advocacy networks, disease education, and specialized support resources.

Trends in the data suggested changing needs and experiences of RTT individuals as they progress from childhood into adolescence and adulthood. This was further evidenced by caregiver interviews, which frequently highlighted challenges associated with this transition. Caregivers reported that the impact of symptom burden increases with age, and time spent on caregiving was highest for adults and lowest for children. As such, developing structured management plans that anticipate changing needs and ensure continuity of care may be effective in ensuring consistently appropriate care.

The fragmentation of services and lack of coordination between HCPs may exacerbate caregiver stress and hinder effective disease management. The number and type of providers involved varied widely, often without a central coordinating clinician. Only 3% of RTT individuals were seen by a sleep specialist, despite sleep disturbance being widely reported as among the most disruptive features of daily life for both individuals with RTT and their caregivers. Critically, sleep disturbances in RTT do not occur in isolation; they interact with epilepsy and broader neurological instability in ways that can exacerbate overall symptom burden and reduce the effectiveness of other interventions [26–28]. Improved access to sleep specialists may therefore represent an opportunity to address a driver of wider neurological and caregiver burden.

Issues consistently highlighted here and elsewhere may inform clinical decision-making and future patient management strategies. A 2015 study suggested that the median time to diagnosis is 2.7 years for classic RTT and 3.8 years for atypical RTT [9]. However, improvements in genetic screening have now increased the potential for timely diagnosis. Still, caregivers consistently highlighted difficulties in obtaining an accurate diagnosis as a significant barrier to accessing care. Improving the availability of genetic testing and incorporating RTT markers into broader genetic screening panels, as currently seen in the Netherlands, may facilitate more timely and accurate diagnoses.

Raising awareness of RTT symptoms among pediatricians, neurologists, and other frontline HCPs could reduce misdiagnoses, shorten diagnostic delays, and support earlier initiation of appropriate care. HCPs’ self-reported capability in managing rare disease patients directly limits implementation, leading to failures or delays in prescribing recommended therapies [29]. A 2017 European Organisation for Rare Diseases (EURORDIS) survey found that nearly a quarter of rare disease patients in Europe did not receive treatment due to a lack of availability [30]. As such, expanding expertise and HCPs able to prescribe RTT treatments when they become available may be desirable to ensure their effective implementation.

Currently, in the absence of approved therapies in Europe, patient management approaches remain central to reducing BOI and improving QOL. It has previously been reported that multinational programmes are likely to have a greater impact on rare diseases than single-country programmes [31], highlighting the urgent need for broader, global reform of care for individuals with Rett syndrome. Improved diagnostic pathways, greater caregiver support, and better coordination of care may offer opportunities to alleviate caregiver strain and improve outcomes for RTT individuals. Beyond RTT, these findings also underscore the broader systemic challenges faced by families navigating rare and under-recognized conditions. Policy initiatives and healthcare system reforms that prioritize rare diseases, support multidisciplinary care, and invest in long-term management infrastructures are urgently needed.

## Conclusions

The challenges identified in this study are consistent with those reported in existing literature, and are contextualized here within a multinational European framework, offering cross-national confirmation. Across eight European countries, caregivers reported consistent unmet needs, high symptom burden, and substantial caregiving demands for individuals with Rett syndrome. Fragmented care, diagnostic delays, and limited coordination between healthcare providers were common challenges that intensified caregiver strain and impeded effective management. As participants were recruited through patient advocacy groups, unmet needs across the broader RTT caregiver community may be greater among families with less access to support resources and specialized care networks. Establishing earlier diagnosis, improving clinical awareness, and developing coordinated, multidisciplinary care pathways may help to reduce burden and support better long-term outcomes, especially during the transition to adulthood. As new treatments emerge, strengthening care infrastructures will be important in ensuring meaningful improvements in quality of life for RTT individuals and their families.

## Data Availability

The data that support the findings of this study are available from Charles River Associates International, but restrictions apply to the availability of these data, which were used under license for the current study, and so are not publicly available. Data are, however, available from the authors upon reasonable request and with permission of Charles River Associates International. Please contact tom.pulles@acadia-pharm.com for such requests.

## Abbreviations

RTT: Rett syndrome
QOL: Quality of life
BOI: Burden of illness
HCP: Healthcare professional
AAC: Augmentative and Alternative Communication
IGF-1: Insulin-like Growth Factor 1
MECP2: Methyl-CpG-binding protein 2
CDKL5: Cyclin-dependent kinase-like 5
FOXG1: Forkhead box G1
